# Significance bias: an empirical evaluation of the oral health literature

**DOI:** 10.1186/s12903-016-0208-x

**Published:** 2016-05-05

**Authors:** Edwin Kagereki, Joseph Gakonyo, Hazel Simila

**Affiliations:** Department of Periodontology/Community and Preventive Dentistry, University of Nairobi, P.O. BOX 30197-00100, Nairobi, Kenya; Department of Conservative and Prosthetic Dentistry, University of Nairobi, P.O. BOX 30197-00100, Nairobi, Kenya

**Keywords:** *P*-value, File drawer effect, Data-dredging, Evidential value, Significance bias

## Abstract

**Background:**

The tendency to selectively report “significant” statistical results (file-drawers effect) or run selective analyses to achieve “significant” results (data-dredging) has been observed in many scientific fields. Subsequently, statistically significant findings may be due to selective reporting rather than a true effect. The p-curve, a distribution of *p*-values from a set of studies, is used to study aspects of statistical evidence in a scientific field. The aim of this study was to assess publication bias and evidential value in oral health research.

**Methods:**

This was a descriptive and exploratory study that analysed the *p*-values published in oral health literature. The National Library of Medicine catalogue was searched for journals published in English, indexed in PubMed and tagged with dentistry Medical Subject Headings (MeSH) words. Web scraping for abstracts published between 2004 and 2014 was done and all *p*-values extracted. A p-curve was generated from the *p*-values and used for analysis. Bayesian binomial analysis was used to test the proportion of the *p*-values on either side of the 0.05 threshold (test for publication bias) or the 0.025 threshold (test for evidential value). The tacit assumption was that significant *p*-values reported were the result of publication bias.

**Results:**

The present study found the use of *p*-values in a total of 44,315 *p*-values published in 12,440 abstracts. Two percent of the *p*-values were inaccurately reported as zero or ≥1. The p-curve was right skewed, with an intriguing bi-modality. The distribution of the *p*-values is also unequal on either side of 0.025 and 0.045 of the p-curve.

**Conclusions:**

This study found evidence of data-dredging, publication bias and errors in the dental literature. Although the present study was conducted on abstracts, the findings highlight a subject that should be researched in future studies that would consider the various factors that may influence *p*-values.

**Electronic supplementary material:**

The online version of this article (doi:10.1186/s12903-016-0208-x) contains supplementary material, which is available to authorized users.

## Background

Goodhart’s law states that “When a measure becomes a target, it ceases to be a good measure” [1]. Prevailing evidence in scientific publications corroborates this law, with many journals selectively publishing statistically significant results [2, 3]. Publication bias is a phenomenon that arises when statistical significance strongly influences the chances of publication. With the ever-increasing pressure to publish or perish, researchers start considering bending the rules to increase the chances of their work getting published [4].

A notable negative effect of publication bias is the influence it has on meta-analysis [5]. The latter combines the quantitative evidence from related studies to summarize a whole body of research on a particular question which is the guiding principle in evidence based medicine. It therefore follows that if the published research findings are biased, then the conclusions drawn might be flawed. A recent study done in Yale claimed to show evidence of an association between dental x-rays and intracranial meningioma [6]. However, upon further interrogation of the study, irreconcilable data problems highlighted serious flaws in the study that render the conclusions invalid [7]. Publication bias also affects the effectiveness of replication as a tool of validation of scientific findings [8]. This bias has been widely studied in the context of null hypothesis significance testing (NHST) whereby the pre-dominant measure of the scientific decisions is the *p*-value. The role of NHST has been questioned on epistemological reasons, with some authors suggesting the abandonment of *p*-values [9, 10]. Some journals like *Epidemiology* [11] and *Basic and applied psychology* [12] have taken a principled stand against them.

The NHST was introduced by R. A. Fisher, Jerzy Neyman and Egon Pearson and has since been widely adopted as the “gold standard” in hypothesis testing [13]. The probability of getting an outcome from the null hypothesis that is as extreme as (or more extreme than) the actual outcome, is called the *p*-value. If the *p*-value is very small, conventionally less than 5 %, then the null hypothesis is rejected. This arbitrary cut-off has led to the scientifically dubious practice of regarding “significant” findings as more valuable, reliable, and reproducible [14]. In reality, there can be many possible *p*-values for any set of data; depending on how and why the data was generated [15]. Furthermore, *p*-values also depend on the tests that the analyst decides to use, making them highly subjective [16]. Thus *p*-values present fundamental logical problems which are highlighted below to induce the readers’ curiosity.

To begin with, the significance tests are often misunderstood and misinterpreted [17]. For example, it is often equated with the strength of a relationship, but a tiny effect size can have very low *p*-values with a large enough sample size. Similarly, a low *p*-value does not mean that a finding is of major clinical or biological significance [18]. Subsequently a *p*-value alone does not reveal relevant information concerning effect sizes and or even the direction of the effect. It is therefore advisable that *p*-values are interpreted in context.

In addition, the analyst has an option to apply alternative methods and tests to get intended results (usually statistically significant findings) without a prior analysis plan to answer the scientific question at hand [16]. In this way the analyst is able to control the false alarms on the basis of his/her intention, not on the basis of the research problem. This debate may continue for a long time, as it touches on philosophy of science.

Researchers have studied various methods in which publication bias has been perpetrated. One such method is data-dredging (also termed as snooping, fishing, significance-chasing or double-dipping) [19]. This entails multiple attempts at data analysis to achieve desired results. For example, an analyst may use partial data to decide whether to or not to continue with the analysis. It may also involve manipulation of variables post-analysis to achieve desirable and pre-determined results [16]. For instance dropping outliers, splitting or regrouping treatment groups or variable transformation. Another way in which publication bias may arise is the ‘file-drawer effect’. This is a phenomenon in which researchers tend to forward studies with significant results for publication, while withholding those with non-significant findings [19].

A p-curve is the distribution of *p*-values for a set of studies which assumes that the distribution of *p*-values is a random variable with some level of uncertainty [20]. This set of *p*-values can form a probability distribution with all possible outcomes and their corresponding probabilities. Thus in reality, the candidate *p*-values form a finite continuum from zero to one, both zero and one being excluded. This curve has been adopted as a tool in the study of evidence in various scientific fields [19, 21].

One application of the p-curve is to detect presence of publication bias. A sharp drop of the p-curve for values above the significance level illustrates this bias [18]. This curve may also be used to detect data-dredging. Here, the assumption is that if researchers turn a nonsignificant *p*-value into a significant one, then the shape of this curve will be altered around the perceived significance threshold [14, 17].

Moreover, the p-curve has been used to study evidential value in a set of studies [14, 17]. This is considered to be present when the published evidence for a specific hypothesis consistently suggests that the effect truly exists across a set of studies. When the true effect is strong, researchers are more likely to obtain very low *p*-values (*p* < 0.001) than moderately low *p*-values (*p* < 0.01), and less likely to obtain non-significant *p*-values (*p* > 0.05) [18]. Therefore, as the true effect size increases the p-curve becomes more skewed to the right [19]. Binomial tests have previously been used to assess existence of evidential value and data-dredging [14, 17]. To achieve this goal, the significant *p*-values are binned into two groups; 0 < *p* ≤ 0.025 (lower bin) and 0.026 ≤ *p* ≤0.05 (upper bin). The assumption here is that if evidential value is present, the expected number of *p*-values in the lower bin should be equal to or greater than that in the upper bin. Conversely, if there are more *p*-values in the upper bin, then data-dredging is a plausible explanation [21].

It has however been noted that the method proposed above only detects severe data-dredging but may fail to detect modest levels [18]. A more sensitive approach would be to narrow down on the *p*-values close to 0.05 where it is expected that the signals of data-dredging would be strongest. It has been established that p-hackers have limited ambition and tend to alter only the *p*-values close to the 0.05 threshold [15]. To do this the *p*-values close to 0.05 are divided into two bins, one between 0.04 and 0.045 (lower bin), and the upper bin to contain *p*-values between 0.046 and 0.05. Ideally the two bins should be equal if there is no manipulation of the *p*-values. Comparing the proportion of the *p*-values in the upper bin to those in the lower bin is a more sensitive test of data-dredging [17].

A subtle technique observed in data-dredging is strategic rounding-off [18, 22]. In this, *p*-values with two to three decimal places above the threshold are conveniently rounded-down to achieve the statistically significant threshold. For instance, if the obtained value is below 0.054 then it is rounded-down to 0.05. To test the presence of this strategic rounding-off, the proportion of marginally significant *p*-values (*p*-values between 0.045 and 0.049) are compared with the marginally non-significant *p*-values (*p*-values between 0.051 and 0.054). It therefore follows that if the marginally non-significant *p*-values are fewer than the marginally significant *p*-values, then there is evidence of strategic rounding off.

The p-curve therefore is a useful tool to help researchers in a field to assess possible ways in which *p*-values could be dragging scientific processes down by biased reporting of the results [23]. The aim of this study was to assess file-drawer effect, data-dredging, strategic rounding-off and evidential value in oral health literature by studying the p-curve. The tacit assumption here was that these factors affect the reported *p*-values. It is hoped that the findings will contribute to the debate on the alternative methods to the NHST.

## Methods

A descriptive and exploratory study analysed the *p*-values published in oral health literature from January 2004 through December 2014. Web scraping for the abstracts published in all the volumes was done and all the *p*-values extracted. A total of 31 journals out of an initial 789 entries were used for the analysis.

### Search strategy

The National Library of Medicine (NLM) catalogue was searched for journals published in English, indexed in PubMed and tagged with dentistry MeSH (Medical Subject Headings) words (MeSH Unique ID: D003813). This search was done with the NLM Catalog Advanced Search Builder using the MeSH word for the entries on “MeSH Major topic” OR “MeSH Terms” OR “MeSH Subheading”. Filters activated were: “Only PubMed journals” and “English”.

**Fig. 1:**
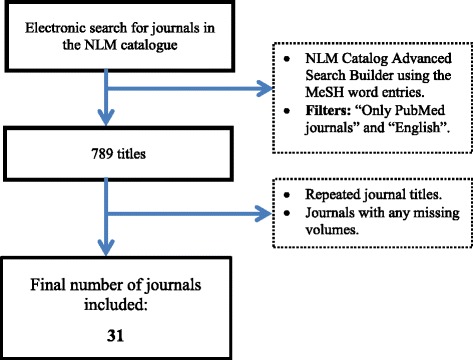
Search strategy. The National Library of Medicine (NLM) catalogue was searched for journals published in English, indexed in PubMed and tagged with dentistry MeSH (Medical Subject Headings) words (MeSH Unique ID: D003813). Repeated entries and journals with missing volumes within the study period were excluded

The results of the search were collected in a collection file and downloaded as a comma separated value (.csv) file. A total of 789 entries were identified. All the duplicated entries and journals missing with any missing volume within the study period were excluded. This is summarized in Fig. 1. All the researchers were involved in the search any arising disputes was resolved through a consensus by all of them.

### Journals included

The following journals were included in this study:

- *J Contemp Dent Pract,Br J Oral Maxillofac Surg,Int J Oral Maxillofac Surg,J Clin Dent,Int J Dent Hyg,BMC Oral Health,Oral Health Prev Dent,Community Dent Oral Epidemiol,J Oral Sci,Braz Oral Res,J Adhes Dent,J Clin Pediatr Dent,J Craniofac Surg,Am J Dent,Community Dent Health,Gerodontology,J Oral Maxillofac Surg,Int Endod J,Eur J Orthod,J Oral Implantol,Gen Dent,J Endod,J Clin Periodontol,J Dent,J Periodontol,Caries Res,J Periodontal Res,Arch Oral Biol,J Prosthet Dent,Int Dent J,Br Dent J,Angle Orthod, Clin Implant Dent Relat Res,Int J Dent Hyg and Oral Health Prev Dent.*

### Statistical analysis

The following variables were collected: title of the journal, PubMed ID, year of publication, *p*-values, title of article and the abstract using *R package* (Version 3.1.2, R Development Core Team, Vienna, Austria). All the data analysis was done using R and the relevant packages. The R code used is provided as Additional file 1.

To test for the distribution of the *p*-values across the thresholds, Bayesian binomial test was used to estimate the 95 % high definition intervals (HDI) estimated. A non-informative prior was used based on the distribution of Beta (1, 1) distribution.

The study examined all the *p*-values that reported in abstract of all the selected journal volumes between 2004 and 2014. However, the *p*-values erroneously reported as zero or one and were excluded from the p-curve analysis. A summary of the research process is depicted in Fig. 1.

## Results

### Number of reported *p*-values

**Fig. 2:**
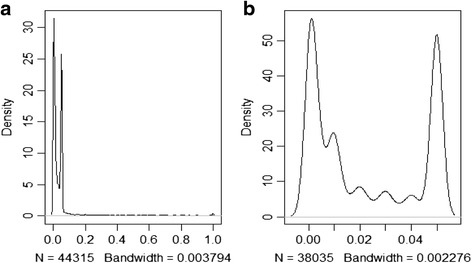
The p-curve of the 44,315 *p*-values studied. The curve on the left **a** illustrates the overabundance of the *p*-values below the 0.05 threshold. The curve on the right **b** is a closer look at the *p*-values below the 0.05 threshold illustrating a bi-modal distribution of the *p*-values; one peak close to zero and the other close to the conventional significant threshold of 0.05

In this study, a total of 44,315 *p*-values were abstracted from 12,440 abstracts. The paper with the maximum number of *p*-values had 48, with most of the other papers reporting a single *p*-value. There were 157(<1 %) *p*-values reported as one and 617(1 %) *p*-values reported as zero. Further, 10,960 (25 %) *p*-values were reported as exactly 0.05. The distribution of the reported *p*-values is summarized in Fig. 2.

### Assessment of the p-curve for selection bias/file-drawer effect

There was an over-abundance of *p*-values below the 0.05 threshold as illustrated in Fig. 2a. A bi-modality was observed in the distribution of all *p*-values: around 0 and also around the significance threshold of .05 as shown in Fig. 2b.

### Assessment of the p-curve for data-dredging and evidential value

To test for evidential value the proportion of the *p*-values below the 0.05 threshold were divided into two bins. There were 22,468 *p*-values in the lower bin (0–0.025), while 15,414 *p*-values were in the upper bin (0.026-0.05). Bayesian binomial test was used to test equality of these proportions. The estimated percentage of the lower *p*-values (up to 0.025) was 59.3 % [58.8, 59.5]. The relative frequency of the lower *p*-values was more than 0.5 estimated by a probability of >0.999 and less than 0.5 by a probability of <0.001.

To test for data-dredging, the *p*-values close to the 0.05 threshold were divided into two bins. There were 1224 *p*-values in the lower bin (0.04-0.45) and 15,414 *p*-values in the upper group (0.046-0.05). Bayesian binomial test was used to test the equality of these proportions. This resulted in an estimated proportion of 0.097 [0.092, 0.102] for the lower bin. The relative frequency of the lower bin was more than 0.5 by a probability of <0.001 and less than 0.5 by a probability of >0.999.

### Strategic rounding of p-values to show significance in reported results

A comparison between the proportion of the marginally significant *p*-values (*p*-values between 0.040 and 0.049) and the proportion of the marginally non-significant *p*-values (*p*-values between 0.051 and 0.054) was done. The marginally significant *p*-values were 15,334 (99.19 %) as compared to the marginally insignificant *p*-values 125 (0.81 %). Bayesian binomial to test the difference between these two proportions estimated the proportion of the marginally significant to be 0.992 [0.99, 0.993]. The relative frequency of the marginally significant was more than 0.5 by a probability of >0.999 and less than 0.5 by a probability of <0.001.

### Reported p-values across the various disciplines in dentistry

A total of 13 dental specialties were considered in this study. This was guided by the MeSH for each journal. Consistently, significant *p*-values were reported across the disciplines. Data-dredging was evident in all the disciplines although dental materials had proof of evidential value (Table 1).

**Table 1:** Tests for evidential value and data-dredging across dental specialties. Evidence of data-dredging was there across the disciplines

Discipline	Frequency	0 to 0.025	0.026-0.05	Test for evidential value	0.04-0.045	0.046-0.05	Test for data-dredging
General Dentistry	10948 (25 %)	5366	4108	0.57 [0.62, 0.64]	212	3364	0.059 [0.052, 0.067]
Surgery	8605 (19 %)	4372	2564	0.63 [0.62, 0.64]	348	1523	0.19 [0.17, 0.20]
Public Health Dentistry	1805 (4 %)	1122	478	0.70 [0.68, 0.72]	62	315	0.17 [0.13, 0.20]
Dental Materials	821 (2 %)	325	392	0.45 [0.42, 0.49]^‡^	1	355	0.0046 [0.00015, 0.013]
Pedodontics	490 (1 %)	246	184	0.57 [0.53, 0.62]	22	114	0.17 [0.11, 0.23]
Gerodonlogy	922 (2 %)	445	316	0.58 [0.55, 0.62]	26	223	0.11 [0.071, 0.15]
Endodontics	5456 (12 %)	2309	2468	0.48 [0.47, 0.50]	109	2133	0.049 [0.040, 0.058]
Orthodontics	2265 (5 %)	1229	736	0.63 [0.60, 0.65]	80	545	0.13 [0.10, 0.16]
Implantology	553 (1 %)	267	170	0.61 [0.57, 0.66]	19	113	0.15 [0.091, 0.21]
Periodontics	8770 (20 %)	4666	3048	0.60 [0.59, 0.62]	298	2074	0.13 [0.11, 0.14]
Cariology	945 (2 %)	565	280	0.67 [0.64, 0.70]	24	189	0.12 [0.075, 0.16]
Oral Hygiene	438 (1 %)	242	146	0.62 [0.58, 0.67]	16	89	0.16 [0.091, 0.23]
Prosthodontics	2231 (5 %)	1311	493	0.73 [0.71, 0.75]	33	338	0.09 [0.062, 0.12]

^‡^The only specialty with evidential value was dental materials

## Discussion

In studying the p-curve we observed that it was generally right skewed with two peaks: one close to 0 and the other near the significance threshold of 0.05. One possible explanation of this finding is based on the general assumption that researchers manipulate their findings to increase chances of their work getting published (strategic reaction to publication bias). The high number of - small *p*-values (less than 0.05) observed in the present study across the range of the oral health specialties (with the exception of dental materials) could also imply that a majority of researchers predominantly study phenomena where an actual difference is known to already exists (evidential value) [20, 24]. It is therefore necessary to conduct further investigations on the research questions studied in oral health.

Statistical power considerations associated with statistical tests of hypotheses relate to the likelihood of correctly rejecting the tested hypotheses, given a particular beta level, alpha level, effect size and sample size. Consequently, an intimate relationship between these four measures exists. Small *p*-values would therefore result from small study effects, large samples, high power or a combination of them. It is therefore possible that these factors may lead to the right skew of the p-curve. Future research is necessary to investigate the foregoing and to secure more evidence on the prevalence of congruence errors in oral health literature [5].

The authors noted that in some of the journal articles included in the present study reported multiple *p*-values suggesting that multiple hypotheses were tested simultaneously. Testing several independent null hypotheses and maintaining the threshold at 0.05 for each comparison, the chance of obtaining at least one “statistically significant” result is greater than 5 % (even if all null hypotheses are true). Conventionally, where multiple testing is done, additional adjustments are done to alleviate the critical values for the hypotheses of interest, and make rejection of these hypotheses more likely. Therefore without further analysis, it is not possible to disregard the possibility that failure to compensate for multiple comparisons could have resulted in the over-abundance in the small *p*-values [25].

The distribution of the *p*-values also, suggests evidence of data-dredging. The higher proportion of the *p*-values close to the 0.05 threshold may suggest that the researchers may have manipulated the *p*-values to get close to the threshold. The results of the present study are in accordance with the majority of the previous findings in other scientific fields [3, 20]. The un-equal distribution of the *p*-values between 0.045 and 0.049 as compared to those between 0.051 and 0.055 could probably be due to the rounding down of the values between 0.051 and 0.054 to achieve the significance value of 0.05 [17].

The analysis presented in this study is rather novel and exploratory and may contribute to the discussion whether we should substantially change the way we do statistics. Further they support the suggestion that many research findings maybe false [2]. On a wider scope, these findings raise many questions on the evidence reported in oral health. One such inquiry is whether there is congruence between the power, effect size, *p*-value and test statistic or repetition of the research hypotheses. Further, one may wish to know if there exists bias where other inferential methods have been used.

## Conclusion

This study found presence of evidential value, data-dredging, publication bias in the oral health literature. The fact that researchers may wish to publish their significant findings in their abstracts while leaving the non-significant results is an inherent limitation of the present study. Additionally, the numerous small *p*-values observed may be attributed to multiple testing. The foregoing can be overcome in future studies by including the full research papers. With the original data, a re-run of all tests would reveal presence of bias where other inferential methods have been used and also identify incongruences in the statistical evidence reported.

## Additional file

Additional file 1: Functions to scrape P-values from Pubmed abstracts. (TXT 5 kb)
